# The rate of screw misplacement in segmental pedicle screw fixation in adolescent idiopathic scoliosis: The effect of learning and cumulative experience

**DOI:** 10.3109/17453674.2010.548032

**Published:** 2011-02-10

**Authors:** Kasim Abul-Kasim, Acke Ohlin

**Affiliations:** ^1^Division of Neuroradiology, Lund University, Diagnostic Centre for Imaging and Functional Medicine, Skåne University Hospital; ^2^Department of Orthopaedic Surgery, Lund University, Skåne University Hospital, Malmö, Sweden

## Abstract

**Background and purpose:**

There are no reports in the literature on the influence of learning on the pedicle screw insertion. We studied the effect of learning on the rate of screw misplacement in patients with adolescent idiopathic scoliosis treated with segmental pedicle screw fixation.

**Method:**

We retrospectively evaluated low-dose spine computed tomography of 116 consecutive patients (aged 16 (12–24) years, 94 females) who were operated during 4 periods over 2005–2009 (group 1: patients operated autumn 2005–2006; group 2: 2007; group 3: 2008; and group 4: 2009). 5 types of misplacement were recorded: medial cortical perforation, lateral cortical perforation, anterior cortical perforation of the vertebral body, endplate perforation, and perforation of the neural foramen.

**Reslts:**

2,201 pedicle screws were evaluated, with an average of 19 screws per patient. The rate of screw misplacement for the whole study was 14%. The rate of lateral and medial cortical perforation was 7% and 5%. There was an inverse correlation between the occurrence of misplacement and the patient number, i.e. the date of operation (r = –0.35; p < 0.001). The skillfulness of screw insertion improved with reduction of the rate of screw misplacement from 20% in 2005–2006 to 11% in 2009, with a breakpoint at the end of the first study period (34 patients).

**Interpretation:**

We found a substantial learning curve; cumulative experience may have contributed to continued reduction of misplacement rate.

Since its introduction by Suk et al. (1995), segmental pedicle screw fixation in adolescent idiopathic scoliosis (AIS) has undergone continuous development. The use of pedicle screws in the thoracic spine is not without risk, however, especially for neurovascular compromise. The rate of neurovascular complications associated with pedicle screw misplacement varies from 0% to 1.3% (Liljenqvist et al. 1997, Belmont et al. 2001 Suk et al. 2001, Coe et al. 2006, Upendra et al. 2008). Evolution of the operative techniques since 1995 (Suk et al. 1995, Kim and Lenke 2005, Kuklo et al. 2005, Lonner et al. 2006), our better understanding of the 3-dimensional nature of adolescent idiopathic scoliosis (AIS), and probably the advances in multidetector computed tomography (CT) technology—with the availability of modern CTs that enable dose reduction—have contributed to increasing use of the “pedicle screw-only construct” in the surgical correction of scoliosis.

To our knowledge, there have been no reports in the literature on the learning curve regarding accuracy of screw placement. The rate of misplacement of thoracic pedicle screws, evaluated with CT, varies from about 6% (Di Silvestre et al. 2007) to 50% (Upendra et al. 2008). In all published reports on the accuracy of pedicle screw placement, the figures presented have shown the overall rate of misplacement while possible improvements in the skill of screw insertion over time have not been studied.

We assessed whether a learning curve exists regarding the accuracy of screw placement in patients with AIS who are treated with segmental pedicle screw fixation.

## Patients and methods

### Patient data

The Suk technique was introduced at the Orthopaedics Department of our hospital in the autumn of 2005, after a study visit to Seoul by the senior author (AO). All 116 patients with AIS who underwent posterior corrective surgery with segmental pedicle screw fixation using titanium “all pedicle screw construct” between autumn 2005 and December 2009 were included in the study. The patients were categorized into 4 groups according to the date of operation as follows; group 1: 34 patients operated on from autumn 2005 through 2006; group 2: 21 patients operated on in 2007; group 3: 25 patients operated on in 2008; and group 4: 36 patients operated on in 2009. As the number of patients operated on in the autumn of 2005 was only 7, these were included in the first period with patients operated on in 2006. The mean age of the patients was 16 (12–24) years and 94 were females.

As the primary aim of our study was to evaluate the effect of the learning curve on the rate of accurate screw placement and not the complications of screw misplacement, the study period was limited to the first postoperative clinical follow-up at 8 weeks. The following data were collected from the clinical and the operative records: age, sex, diagnosis, intraoperative events, postoperative events, and the clinical status at 8 weeks. At the latter visit, all patients were asked whether they had any history of neurological symptoms related to medullary or nerve root compromise or evidence of myelopathy. Increased tendon reflexes, abnormal clonus, and Babinski sign were specifically sought for. The radiological data gathered from the pre- and postoperative standing radiographs and CTs were: Cobb angle before and after surgery, Lenke classification, the number of screws in the operated constructs, and the positions of screws. The level and the side of every individual screw as well as the relation to the scoliotic concavity and convexity were also recorded. The relationship of screws to the pedicle, vertebral body, scoliotic apex, concavity/convexity of the curve, and the surrounding structures e.g. aorta, pleura, and the pedicle rib unit (PRU) were recorded.

The use of low-dose CT in the work-up of AIS was approved by the regional radiation protection committee and the study was approved by the regional ethics committee.

### Radiological work-up

Plain radiographs were obtained pre- and postoperatively with low-dose radiation; this included posteroanterior and lateral views. CT examinations were performed with low-radiation-dose spine CT at 6–8 weeks after surgery. All examinations were performed on a 16-slice CT scanner (SOMATOM Sensation 16; Siemens AG, Forchheim, Germany) with the following scan parameters: slice collimation 16 × 0.75 mm, rotation time 0.75 s, pitch 1.5, tube voltage 80 kV, and quality reference for the effective tube current-time product 25 mAs (Abul-Kasim et al. 2009). The effective radiation dose of low-dose CT including 15 vertebral bodies has been reported to be 0.37 mSv (Abul-Kasim et al. 2009a). The slice collimation of 0.75 mm allowed us to obtain 1-mm and 3-mm thick reformatted axial images with soft-tissue algorithm and skeletal algorithm respectively, and 2-mm thick coronal and sagittal reformatted images. Soft-tissue algorithm was used to allow reduction of streak artifacts from the hardware.

### Grading of screw misplacement

**Figure 1.**
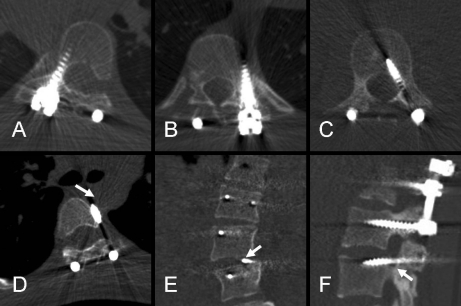
Axial (A–D), coronal (E) and sagittal (F) images obtained by low-dose spine CT from 6 patients. A. Normally-placed screw through the right pedicle of T7. B. Screw with total lateral cortical perforation (LCP grade 2) at the level of T9. C. Screw with total medial cortical perforation (MCP grade 2) at the level of L3. D. Pedicle screw with anterior cortical perforation (ACP) with screw tip in close proximity to the posterior-lateral limit of aorta (arrow). This screw was removed a few months later. E. Screw tip perforating the upper endplate of L4 (arrow) on the left side (EPP). F. Screw passing through the lower boundary of the neural foramen (FP) below L3 (arrow).

The classification of the pedicle screw misplacement was performed according to the recently established and reported grading system based on whether the violation of pedicular cortex was total or partial rather than on measuring the degree of misplacement in mm (Abul-Kasim et al. 2009b, 2010). 5 types of misplacement were recorded, namely medial cortical perforation (MCP), lateral cortical perforation (LCP), anterior cortical perforation of vertebral body (ACP), endplate perforation (EPP), and perforation of neural foramen (FP). The medial and lateral cortical perforations were graded as follows: grade 1 when the pedicle screw was partially medialized or lateralized, and grade 2 when the pedicle screw was totally medialized or lateralized (Figure 1). All examinations were read on 2 different occasions by a senior neuroradiologist experienced in radiology of spinal deformity. In cases of different grading on the 2 occasions of evaluation, a consensus was reached by joint evaluation with another neuroradiologist. The misplacement rate for every individual patient was calculated as the number of misplaced screws times 100 divided by the total number of screws inserted.

### Operative technique

All operations were performed through a standard posterior exposure. The entry points for screws were determined after identification of the bony landmarks. At each assumed entry point, a 3-cm long and 1-mm wide titanium bone marker was inserted. By intermittent exposure with a C-arm fluoroscope in the anteroposterior view, the position and the degree of tilt of the bone marker were estimated. The screw canal was prepared with a hand-driven drill. After drilling, a probe or “feeler” was used to palpate the bottom and borders of the screw canal. Thereafter, self-tapping transpedicular screws were sequentially introduced. Curve correction was performed by simple rod derotation as well as direct vertebral rotation (DVR) when having only the concave rod in place (Lee et al. 2004). The posterior elements were decorticated, and finally the contoured stabilizing rod was inserted. All operations were performed under spinal cord monitoring by motor-evoked potential (MEP). The implants used were made of titanium alloy (EXPEDIUM Spine System). Screws were of uniplanar type with a diameter of 4–6 mm, depending on the pedicular width.

### Statistics

Estimates are presented as mean (SD). Sampling uncertainty of estimates is presented as the 95% confidence interval (CI). Possible correlation between the operation date (i.e. the number of the patient) and the misplacement rate was also tested. Student t-tests were performed to find out whether there were breakpoints in the learning curve that defined the maximal learning by comparing the rate of misplacement: (a) in patients operated on during the first period with that in patients operated on during the remaining study periods (2007–2009), and (b) in patients operated on during the first 2 periods (2005–2007) with that in patients operated on during the last 2 periods (2008–2009). Chi-square test was performed to test the association of the occurrence of screw misplacement with different categorical variables (sex, side, level of scoliotic apex, and scoliotic convexity/convexity). Differences with p-values of < 0.05 were considered statistically significant. We used SPSS software version 17 for all statistical analyses.

## Results

### Characteristics of curve

The distribution of different types of scoliotic curves according to the Lenke classification was as follows (n/%): type 1 (60/52), type 2 (7/6), type 3 (19/16), type 4 (5/4), type 5 (15/13), and type 6 (10/9). The Cobb angle before and after surgery was 56° (SD 10) and 17° (SD 8), which means an angle reduction by 38° (SD 9) (69%) (p < 0.001).

### Rate of screw misplacement

We evaluated 2,201 pedicle screws (116 patients with an average of 19 screws per patient). The mean rate of screw misplacement for the whole study population operated on between the autumn of 2005 and 2009 was 14 (median 13). The rate of misplacement was zero in 16 patients (14%). The rate of lateral and medial cortical perforation was 7% and 5% (Table 1). 1 patient reported postoperative pain and paresthesia in a specific dermatome distribution (T8–T10 on the right side). Low-dose CT showed no medial cortical perforation or foraminal perforation that could explain the neurological deficit. 11 of 18 screw tips that ended in the vicinity of the aorta (1–4 mm) were inserted in 2005–2006 (p < 0.001). The number of laterally placed screws that passed through the pedicle rib unit (using the in-out-in screw technique) increased successively from 3 in 2005–2006 to 9 in 2009 (p < 0.001). No single pedicular fracture related to the correction maneuver was observed.

**Table 1.** Rate of different types of screw misplacement assessed in the study

	2005–2009	Period 1	Period 2	Period 3	Period 4
LCP1	75	20	12	18	25
LCP2	82	38	13	14	17
LCP, total	157 (7)	58	25	32	42
MCP1	67	24	13	10	20
MCP2	44	17	7	9	11
MCP, total	111 (5)	41	20	19	31
ACP	11	4	4	1	2
EPP	11	6	0	3	2
FP	11	5	1	2	3
Combination of different types **^a^**	14	4	2	4	4
Total rate of misplacement	315	118	52	61	84
Misplacement rate per patient **^b^**	14	20	13	12	11
Total number of screws	2201	586	383	493	739
Total number of patients	116	34	21	25	36
Screws per patient	19	17.2	18.2	19.7	20.5

**^a^** Combination of different types: includes combinations of LCP and 1 of the other types, namely ACP, EPP, or FP. Figures in parentheses represent percentage.

**^b^** Misplacement rate per patient (%) equals the number of misplaced screws x 100 divided by the total number of screws inserted.

2 patients were reoperated for removal of misplaced pedicle screws: 1 with ACP with the screw tip in close proximity to the aorta to prevent direct vascular injury or development of pseudoaneurysm, and 1 with MCP and 6–7 mm of spinal canal encroachment.

### Rate of screw misplacement during different study periods

**Figure 2.**
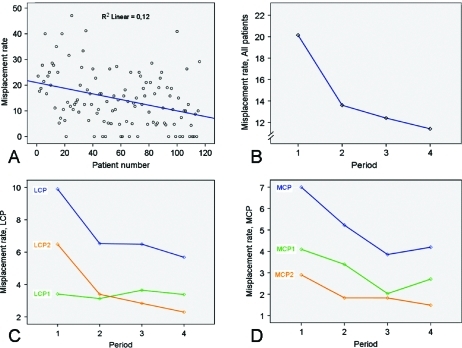
A. Scatter plot showing the results of the linear regression: patient number (date of operation) against the misplacement rate (R2 = 0.12). B–D. Line graphs showing the misplacement rate for different study periods: B. Misplacement rate for all types of misplacement. C. Rate of lateral cortical perforation (all LCP, LCP grade 1, and LCP grade 2). D. Rate of medial cortical perforation (all MCP, MCP grade 1, and MCP grade 2). Periods 1–4 indicate patients operated during 2005–2006, 2007, 2008, and 2009, respectively.

We observed a statistically significant inverse correlation between the occurrence of misplacement and the patient number i.e. the date of operation (correlation coefficient of –0.35; p < 0.001) (Figure 2A). The skillfulness of screw insertion improved significantly, with reduction of the rate of screw misplacement from 20% in 2005–2006 to 11% in 2009. The same applied to the rate of lateral and medial cortical perforation (Table 1 and Figure 2). The rate of medial cortical perforation increased slightly, however, from 3.9% in 2008 to 4.2% in 2009, which mainly depended on increase in grade-1 medial cortical perforation.

We found a difference between the rate of misplacement per patient operated on during the first period of the study (20%) and the rate of misplacement per patient operated on during the remaining 3 periods (12%) (p < 0.001). This also applied to lateral cortical perforation with a mean difference of 3.4% (p = 0.01). The mean difference between the rate of medial cortical perforation in the first period and the 3 remaining study periods was 2.8% (p = 0.05) (Table 2). Corresponding values for differences in the misplacement rate in patients who were operated in the first 2 periods and the second 2 periods were 2.2% (p = 0.08) for lateral cortical perforation and 2.4% (p = 0.07) for medial cortical perforation.

**Table 2.** Rate of misplacement per patient: Student's t-test to find out if there was a breakpoint at the end of the first period, i.e. at the end of 2006 compared with the period 2007–2009

Type of misplacement	Period 1	Period 2–4	Mean difference	95% CI	p-value
Total	20	12	8	3.9–12	< 0.001
LCP	9.6	6.2	3.4	0.7–6.1	0.01
MCP	7.1	4.3	2.8	–0.0001–5.6	0.05

### Association between screw misplacement and different study variables

No statistically significant correlation was found between the patient's age and the rate of misplacement (correlation coefficient –0.10; p = 0.3). No statistically significant association was found between the occurrence of screw misplacement and sex, concavity of the scoliotic curvature, or the side of the apex of the major curve. However, the proportion of patients with no screw misplacement was higher in males (6/22 males as opposed to only 10/94 females) (Table 3). The rate of screw misplacement in thoracic pedicles was 14% and it was 10% in lumbar pedicles (p = 0.001). The rate of misplacement was 20% in the upper thoracic levels (T1–T6) and 13% in the lower thoracic levels (T7–T12) (p < 0.001) (Table 3).

**Table 3.** Chi-square test of association between the occurrence of screw misplacement and different categorical variables

Different variables			p-value
Patients with	Male	Female	
optimally placed screws	6	10	
misplaced screws	16	84	0.08
	Male	Female	
Optimally placed screws	384 (88%)	1,502 (85%)	
Misplaced screws	54 (12%)	261 (15%)	0.2
	Convex	Concave	
Optimally placed screws	787 (85%)	1,099 (86%)	
Misplaced screws	134 (15%)	181 (14%)	0.8
	Right	Left	
Optimally placed screws	727 (86%)	1,159 (86%)	
Misplaced screws	120 (14%)	195 (14%)	0.9
	Thoracic	Lumbar	
Optimally placed screws	1,458 (84%)	428 (90%)	
Misplaced screws	269 (14%)	46 (10%)	0.001
	T1–6	T7–12	
Optimally placed screws	537 (80%)	921 (87%)	
Misplaced screws	137 (20%)	132 (13%)	< 0.001

### Association between screw misplacement and type of curve

Although patients with a Lenke type-1 curve showed a higher rate of screw misplacement (16%), no statistically significant correlation was found between the Lenke type and the rate of screw misplacement (p = 0.6). The association between the Lenke type on one hand and the LCP and MCP on the other was not statistically significant (p = 0.9 and p = 0.08). However, the rate of MCP was twice as common in Lenke 1 than in Lenke 3 and 5 (7% vs. 3%) (p = 0.08). The number of fixed levels and screws inserted was highest in patients with Lenke 3, 4, and 6 curves, depending on whether they were double or triple curves (Table 4).

**Table 4.** The number of screws inserted, the number of the level fixed, and the rate of screw misplacement in relation to different types of scoliotic curves (according to the Lenke classification)

Lenke type	Number of levels fixed	Number of pedicle screws inserted	Rate of all types of misplacement	Rate of LCP	Rate of MCP
1	12 (10–15)	18 (14–26)	16 (0–47)	8 (0–41)	7 (0–35)
2	11 (7–14)	17 (13–23)	12 (0–33)	6 (0–16)	4 (0–27)
3	13 (11–15)	22 (19–25)	13 (0–41)	7 (0–20)	3 (0–23)
4	13 (11–15)	21 (17–24)	13 (0–29)	6 (0–13)	5 (0–8)
5	11 (7–13)	20 (14–24)	13 (0–32)	6 (0–21)	3 (0–13)
6	13 (10–15)	21 (16–27)	12 (0–25)	6 (0–14)	5 (0–15)

## Discussion

Although segmental pedicle screw fixation is widely used nowadays to provide anchors for rods in the posterior fixation following correction of the scoliotic deformity in AIS and the rate of neurovascular complication is often less than 1%, the occurrence of such complications should be avoided by all means available. Increased familiarity with the surgical technique and also experience are the most effective means of avoiding screw misplacement.

The learning curve for thoracic pedicle screw insertion has recently been studied regarding improvement of different parameters such as duration of surgery, amount of blood loss, major curve correction, hospital stay, and complication rate (Lonner et al. 2009) but we could not find any studies on the learning curve regarding accurate screw placement. We found an average misplacement rate of 14%, with a clear learning curve. The rate of misplacement dropped from 20% at the end of the first period of the study to 13% at the end of the second period. We believe that the continued reduction of the misplacement rate to 11% at the end of the study period can be attributed to the experience gathered, and increased familiarity with the surgical technique.

The senior surgeon received a yearly report on the misplacement rate from the radiologist evaluating the screw placement. The radiological and medical records of the patients were scrutinized at a joint evaluation in 2007 (the second period of the study), which resulted in measures that we believe also contributed to the continued improvement in misplacement rate during the remaining periods of the study: (1) The choice of the screws adjusted to the pedicle diameters measured at the preoperative low-dose spine CT contributed to a general decrease in the misplacement rate. (2) Shorter screw length used during the last 2 years of the study period resulted in a reduction in anterior cortical perforation from 8 (in 2005–2007) to 3 (in 2008–2009), and subsequently a decrease in the number of screws (with ACP or LCP) whose tip was close to the aorta from 16 (in 2006–2007) to 2 (in 2008–2009). (3) The number of screws inserted using the “in-out-in technique” through the pedicle rib unit increased from 6 during the first 2 periods to 17 during the last 2 periods. We believe that the latter at least partly contributed to reduction of the rate of lateral cortical perforation, as the surgeons intentionally used this technique in thoracic segments with narrow pedicles. The decreasing rate of medial cortical perforation was just above the limit for statistical significance, but this type of misplacement decreased continuously throughout the study period. The slight increase in the last year of the study period was attributed to a slight increase in the rate of partial grade of misplacement (MCP1) whereas the rate of total medial cortical perforation (MCP2) continued to decrease.

1 patient reported a postoperative neurological deficit related to the right-sided T8–T10-dermatome. As no screw misplacement was found at these levels, the neurological deficit was considered to be due to a local extraforaminal nerve injury, which may have been related to some operative injury during preparation before the pedicle insertion. 2 patients (2 screws) were reoperated for removal of misplaced pedicle screws. In our opinion, removal of misplaced pedicles screws should be restricted to: (1) misplaced pedicle screws in patients with neurological deficit corresponding to the level of misplacement, (2) misplaced pedicle screws with screw tip abutting the aorta, and (3) medially misplaced pedicle screws with canal encroachment exceeding the pedicle diameter.

Studies dealing with screw misplacement following posterior spinal fixation with segmental pedicle screw fixation have shown varying degrees of screw misplacement depending on (a) whether only thoracic, lumbar spine, or both were included, (b) the radiological modality used for evaluation of screw insertion, (c) the grading system used during the evaluation, and (d) the peroperative guide used during screw insertion. Misplacement rates as low as 2.7% were reported for screw insertion of the lumbar spine (Schwarzenbach et al. 1997) and as low as 1.5% when screw insertion was evaluated with plain radiography (Suk et al. 2001). Previous studies have, however, shown that CT may reveal a 10 times higher misplacement rate than does plain radiography (Farber et al. 1995). A study using a strict grading system that classified even 1 mm of cortical violation as misplacement reported misplacement rates of up to 50% (Upendra et al. 2008). Screw misplacement was found to be substantially reduced using computer-assisted orthopedic surgery (CAOS) in pedicle screw insertion in a porcine cadaver model (Richards et al. 2007) and using a CT-guided O-arm (Nottmeier et al. 2010). Our reported rate of screw misplacement of 14% for the whole study period (11% for the last period (2009)) is in accordance with most of the reports in the literature for patients with scoliosis (Halm et al. 1996, Liljenqvist et al. 1997, Di Silvestre et al. 2007). However, the total rate of misplacement often overshadows the successive improvement over time—as our study revealed, namely a clear improvement in screw placement during the last year of the study (11%). Our findings validate our previous conclusions about the reliability of low-dose spine CT and our proposed grading system in the evaluation of screw misplacement (Abul-Kasim et al. 2009b, 2010).

As in other surgical fields, training, coaching, team work, feedback to surgeons, and experience are among the most important tools to reduce the rate of misplacement and other complications—as well as reduction of the duration of surgery, blood loss, and hospital stay. Surgical navigation systems such as O-arm providing 2-D and 3-D intraoperative imaging may further reduce the rate of screw misplacement in spinal deformity surgery. However, this technique means increased exposure of young individuals with AIS to radiation (and thus needs to be optimized with regard to radiation dose), increased cost, and extra training of the surgeons and operating staff.
